# Non-completion of secondary education and early disability in Norway: geographic patterns, individual and community risks

**DOI:** 10.1186/s12889-018-5551-1

**Published:** 2018-05-31

**Authors:** Arnhild Myhr, Tommy Haugan, Monica Lillefjell, Thomas Halvorsen

**Affiliations:** 1 Department of Neuromedicine and Movement Science, Faculty of Medicine and Health Sciences, Norwegian University of Science and Technology, Trondheim, Norway; 2 Faculty of Nursing and Health Sciences, Nord University, Levanger, Norway; 3 Department of Health Research, SINTEF Technology and Society, Trondheim, Norway

**Keywords:** Norway, Secondary education, Disability pension, Social inequality, Community characteristics, Geographic information systems, Multilevel

## Abstract

**Background:**

School non-completion and early work disability is a great public health challenge in Norway, as in most western countries. This study aims to investigate how medically based disability pension (DP) among young adults varies geographically and how municipal socioeconomic conditions interact with non-completion of secondary education in determining DP risk.

**Methods:**

The study includes a nationally representative sample of 30% of all Norwegians (*N* = 350,699) aged 21–40 in 2010 from Statistic Norway’s population registries. Multilevel models incorporating factors at the individual, neighbourhood and municipal levels were applied to estimate the neighbourhood and municipality general contextual effects in DP receipt, and detect possible differences in the impact of municipal socioeconomic conditions on DP risk between completers and non-completers of secondary education.

**Results:**

A pattern of spatial clustering at the neighbourhood (ICC = 0.124) and municipality (ICC = 0.021) levels are clearly evident, indicating that the underlying causes of DP receipt have a systematic neighbourhood and municipality variation in Norway. Non-completion of secondary education is strongly correlated with DP receipt among those younger than 40. Socioeconomic characteristics of the municipality are also significantly correlated with DP risk, but these associations are conditioned by the completion of secondary education. Living in a socioeconomically advantageous municipality (i.e. high income, high education levels and low unemployment and social security payment rates) is associated with a higher risk of DP, but only among those who do not complete their secondary education. Although the proportion of DPs was equal in rural and urban areas, it is evident that young people living in urban settings are more at risk of early DP than their counterparts living in rural parts of the country when controlling for other risk factors.

**Conclusion:**

The association between school non-completion and DP risk varies between municipalities and local socioeconomic environments. The interplay between personal characteristics and the local community is important in DP risk among young adults, implying that preventive measures should be directed not only at the individual level, but also include the educational system and the local community.

## Background

The proportion of young adults prematurely leaving the labour market due to disability pensions (DPs) has increased significantly during the last decade [1]. Recent statistics have shown that 1.8% of the Norwegian population between the ages of 18 and 29 receive DP, which is almost double since 2007 [2]. The leading reason for DP receipt among individuals below 40 years of age in Norway is mental illness [3]. Brage and Thune [3] attributed this increase in part to more precise diagnostics, changes in health status and growing requirements in the labour market. Early work-life exit among young people is a great public health challenge and a threat to the Nordic welfare state model, which depends on high employment rates [4]. Young adults who come to rely on social insurance benefits for most of their life course place a high socioeconomic burden on their society [5]. They also experience substantial lifetime consequences in terms of health and socioeconomic marginalisation [6, 7]. Increasing DP rates will, therefore, ultimately lead to a society with larger socioeconomic and health disparities. DP receipt is also an important area for scientific inquiry because DP is an indicator of society’s health status as a whole, given that DP eligibility criteria are strictly medical [8].

Social factors present at different levels of society, at the individual, family, community and national levels, strongly influence the health of young people [9]. Individual factors related to early DP have been extensively investigated, showing a clear educational gradient with heavy clustering of DP among non-completers of secondary education [10, 11]. Low education achievement is associated with lower work participation, higher risk of long-term socioeconomic marginalisation [12–15], unemployment [16] and mental and physical health issues [9, 17, 18]. School non-completers are also far more likely to receive DP [10, 11, 19] or depend on other medical and non-medical public benefits early in life [10, 11, 20]. Moreover, numerous studies have shown that childhood adversities, such as parental disability and low socioeconomic status, are associated with physical and mental health problems [21–27], low educational achievement [28, 29] and work disability [10, 30, 31] later in life.

A large body of research have linked area characteristics, both physical and social, to a range of health behaviours and health related outcomes [32–35]. Official statistics demonstrate, for instance, large geographical variations in DP recipient rates in Norway, and that certain structural (contextual) factors may partly explain this variation [2]. Nordic population studies have shown that the prevalence of DP correlates with municipal socioeconomic conditions, such as economic development, unemployment rate and education level [36–38]. Moreover, socioeconomically disadvantaged areas are associated with fewer health-promoting behaviours [33, 39], higher morbidity [33, 40, 41] and all-cause mortality [42]. A number of studies have examined the effect of local socioeconomic conditions on the incidence of DP receipt, but less attention has been paid to the variation in contextual risk across subgroups of the population. It is plausible that the socioeconomic context of the area may not equally affect health for all people and certain personal characteristics and features of the social environment may act as moderators [34, 43]. In other words, there may be statistical interactions between personal characteristics, features of the residential context and the health outcomes studied. According to the relative deprivation hypothesis, individuals who are disadvantaged, relative to others in a certain neighbourhood, will experience stress-inducing social comparisons, which may have adverse consequences for individual health [34, 44].

This study investigates how medically based DP among young adults varies geographically, and how municipal socioeconomic conditions interacts with non-completion of secondary education in determining DP risk.

The specific aims of the study were:

(i). to explore geographic distributions of non-completion of secondary education and DP among young adults in Norway;
(ii). to assess how neighbourhood and municipality differences relate to DP risk in young adulthood; and.
(iii). to examine whether municipal socioeconomic conditions interact with the association between school non-completion and risk of DP in young adulthood.

## Methods

### Study population

**Fig. 1:**
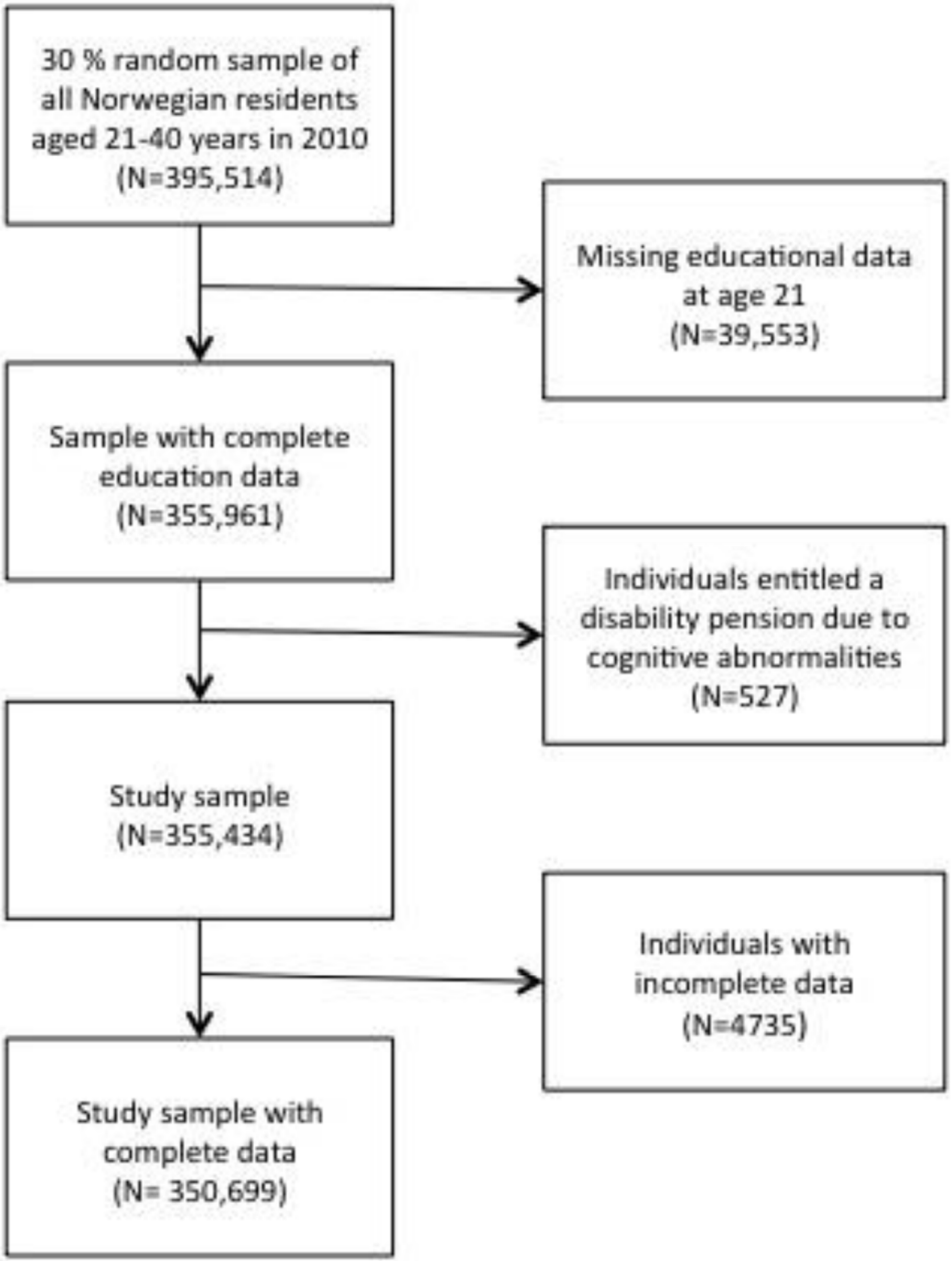
Flow chart of the participants in the present study who where included in the analysis. The proportion of eligible subjects with complete data is 88.7%

This study builds on a 30% random sample, stratified by age, gender and municipality of residence of the entire Norwegian population aged 21–40 years in 2010 (*N* = 395,514), extracted from Statistic Norway’s event database, FD-trygd [45]. These data are linked to the National Education Database (NUDB) through a unique 11-digit personal identification number assigned to all Norwegian citizens. Entitlement of DP was observed at the end of 2010, when respondents were between the ages 21 and 40. The main focus of this study centred on the mechanisms for exclusion from working life. Hence, individuals entitled to a DP due to cognitive abnormalities (*N* = 527) (mainly those with extensive cognitive disabilities), most of whom never achieve ordinary paid work, were excluded from the study. See Fig. 1 for inclusion and exclusion criteria for the present study. The final sample size was 350,699 individuals. The unique personal identification number allowed us to identify information about the individual’s registered parents (or caregivers). We merged the dataset with census information on individuals’ home municipalities, using macro statistics on demography, employment and economic development from the Norwegian Social Science Data (NSD) regional database.

### Measures

#### The outcome variable

Our dependent variable was whether the individual was registered as a DP recipient in the National Insurance Administration (FD-trygd database) at the end of the follow-up period in 2010. In Norway, the eligibility criteria for granting a DP is strictly medical, based on an assessment that a person’s earning ability is permanently reduced by at least 50% due to illness, injury or disability. In addition, the applicants need to meet the following criteria: (1) be between the ages 16 and 67, (2) have been a member of the national insurance program for at least 3 years (all residents of the country are members) and (3) have undergone appropriate medical treatment and rehabilitation that might improve their earning ability.

#### Explanatory variables

##### Individual level

For each individual, we sourced information on age, gender, employment record and parental DP from the FD-trygd database. Parental DP is known to be associated with both low educational achievement and early DP [10, 30] and was, therefore, included as a covariate in the analysis. NUDB provided secondary education data on non-completion, defined as having not obtained a secondary education degree by age 21. The variable was used as both an explanatory and moderator variable in the final analysis. Official statistics [46] have shown that men dominate the 20–29 group of DP recipients, while women are overrepresented in the 30–39 age group. This study accounted for the bias this disparity could present by interacting age with gender in the analysis. In other words, the model reflected the effects of gender at different ages.

##### Neighbourhood level

The FD-trygd database provides information on neighbourhood of residence, which constituted our second level of analysis. We used the individual’s recorded census enumeration district in 2010, which is the lowest geographical level for Norwegian population statistics, to identify their neighbourhoods [45]. The binary variable “rural” identifies the neighbourhood of residence as rural or urban. Urban settlements have clusters of homes where at least 200 people live within a distance of 50 m or less, while the rural areas have a lower population density than this threshold [47].

##### Municipality level

The third unit of analysis comprised all the 430 Norwegian municipalities in 2010. Norway’s municipalities are subject to several common national laws and regulations, which means that they represent relatively homogeneous and, therefore, comparable units. Our model included a spatial lag variable and a set of municipality characteristics that describe the socioeconomic conditions. The spatial lag variable is the mean of the age-adjusted DP rates in neighbouring municipalities and is included to account for spatial dependencies that may exist in the larger regional context. Education level, defined as the percentage of persons aged 20–39 who completed secondary education, and income (the average gross income for all municipal residents aged 17 years and above) were used to evaluate the importance of the municipal socioeconomic environment. The analysis also included the percentage of inhabitants aged 20–39 years receiving unemployment benefits and social security benefits, which reflect the socioeconomic environment more indirectly. The municipality characteristics enter the model as continuous (grand mean centred), 2010 census variables (except the income variable, which was only available for 2009), and are sourced from NSD’s regional database.

### Statistical approach

We investigated the relevance of the residential context as well as the association between municipal socioeconomic conditions and DP receipt in young adulthood and tested the hypothetical interactions using logistic multilevel models [48–50]. Individuals (level 1) are nested within neighbourhoods (level 2, *N* = 12,894), which are nested within municipalities (level 3, *N* = 430). Each of these contexts may condition individual level variation due to unmeasured factors. Hence, we fitted a three-level random intercept model by using maximum likelihood estimation [48–50] to distinguish the individual, neighbourhood and municipality sources of variation in DP receipt. The multilevel framework allows us to simultaneously examine the effects of group-level and individual-level predictors while also accounting for non-independence of observations (clustering) within higher-level units. We modelled the prediction of DP receipt in young adulthood in 10 steps. First, we estimated an “empty” model, only including a random intercept, which represents the variation in DP between the three initial levels. This allowed us to determine the impact of the neighbourhood and municipality context in DP receipt [51]. Models 2–4 in Table 2 included all the individual level variables. In Table 3, we extended the random intercept logit model for the relationship between school non-completion and DP risk to allow non-completion effect to vary across municipalities. Multilevel models with many random components are computationally demanding and, given our large dataset, such models became intractable. Thus, to keep the model simple, a two-level random slope model (i.e. individuals nested within municipalities) was fitted in order to examine whether the relationship between school non-completion and DP risk varies between municipalities. A likelihood ratio test (LR test) was used to compare the random intercept and the random slope model’s goodness of fit. In the final steps, we included all the neighbourhood and municipality variables and adjusted for age, gender and parental DP receipt (Table 4). Models 2–5 added the interaction terms of non-completion of secondary education with the municipality variables: education level (Model 2), gross household income (Model 3), unemployment rate (Model 4) and the rate of social security payments (Model 5).

To quantify the influence of neighbourhood and municipality of residence in DP receipt, we computed the median odds ratio (MOR) [51] and the intraclass correlation coefficients (ICCs) [50]. The MOR translates the area level variance on the log-odds scale and may, in our case, in a simplified way, be interpreted as the increased median odds for receiving a DP if an individual lived in another neighbourhood (or municipality) with a higher risk [52, 53]. Thus, the higher the MOR, the greater the contextual effects. The ICC expresses the correlation in the outcome (i.e. DP receipt) between two individuals taken randomly from the same neighbourhood (or municipality). By using the latent variable method [50, 54], which considers the variance from a standard logistic distribution (π^2^/3 = 3.29), we calculated the ICC with the following formula:

$$ ICC=\frac{Var\left({V}_f\right)}{Var\left({V}_f\right)+\frac{\pi^2}{3}} $$

The percentage of proportional change in variance (PCV) at the respective levels quantifies the percentage of the variance of the empty model explainable by predictor variables inserted into the more complex models [55]. The model parameters were estimated by a mixed effects method using Stata/MP software (version 13). We used geographic information system (GIS) [56, 57] to explore and visualise the geographical patterns of school non-completion and DP receipt among young adults. Measures of global spatial correlation were calculated using the Global Moran’s I statistic and local indicators of spatial association (LISA), which evaluates whether the pattern expressed (i.e. school non-completion and DP receipt) is clustered, dispersed or random [58, 59]. Finally, we used ArcGIS 10 for Desktop for the spatial analysis.

## Results

### Descriptive statistics

Table 1 presents descriptive information for the individual and contextual variables among receivers and non-receivers of DP. At the end of 2010, a total of 7065 (2.0%) individuals were receiving DP, of which 83.2% (*N* = 5876) had not completed secondary education. Of those who received DP, 37% (*N* = 2615) had no previous employment records registered in the FD-trygd database, compared to 2.7% (*N* = 9191) in the non-receiving population.

**Table 1:** Individual and community characteristics of young receivers and non-receivers of disability pension (DP)

Variable	DP receivers (*N* = 7065)		Non-receivers (*N* = 343,634)	
	N	%	N	%
Individual level variables				
Male	3673	52.0	174,696	50.8
Mean Age (SD)	33.4^b^	5.50	30.7	5.92
Secondary education non-completion	5876^a^	83.2	110,719	32.2
Birth cohort				
1970–1974	3204^a^	45.4	94,279	27.4
1975–1979	1798^a^	25.5	81,946	23.9
1980–1984	1180^a^	16.7	80,537	23.4
1985–1989	883^a^	12.5	86,872	25.3
No previous employment records	2615^a^	37.0	9191	2,7
Maternal DP	1544^a^	21.9	32,551	9.5
Mother’s identity unknown	282^a^	4.0	23,570	6.9
Paternal DP	1218^a^	17.2	27,781	8.1
Father’s identity unknown	114^a^	1.6	18,669	5.4
Neighbourhood level variable				
Rural place of residence	1304	18.5	63,435	18.5
Municipality level variables				
Socioeconomic variables	Mean	SD	Mean	SD
Secondary or tertiary education %	66.0^b^	4.64	66.7	4.46
Unemployment %	3.3^b^	0.76	3.2	0.71
Social security benefits %	6.1^b^	2.07	5.7	1.86
Disability benefits %	9.7^b^	2.91	8.7	2.76
Gross household income (thousands)	339.4^b^	382.2	349.8	398.5

^a^Significant difference (*p*-value≤0.05) between groups tested by chi square test ^b^ Significant mean difference (*p*-value≤0.05) between groups tested by independent sample t-test

### Spatial pattern of secondary education non-completion and DP rates among young adults

**Fig. 2:**
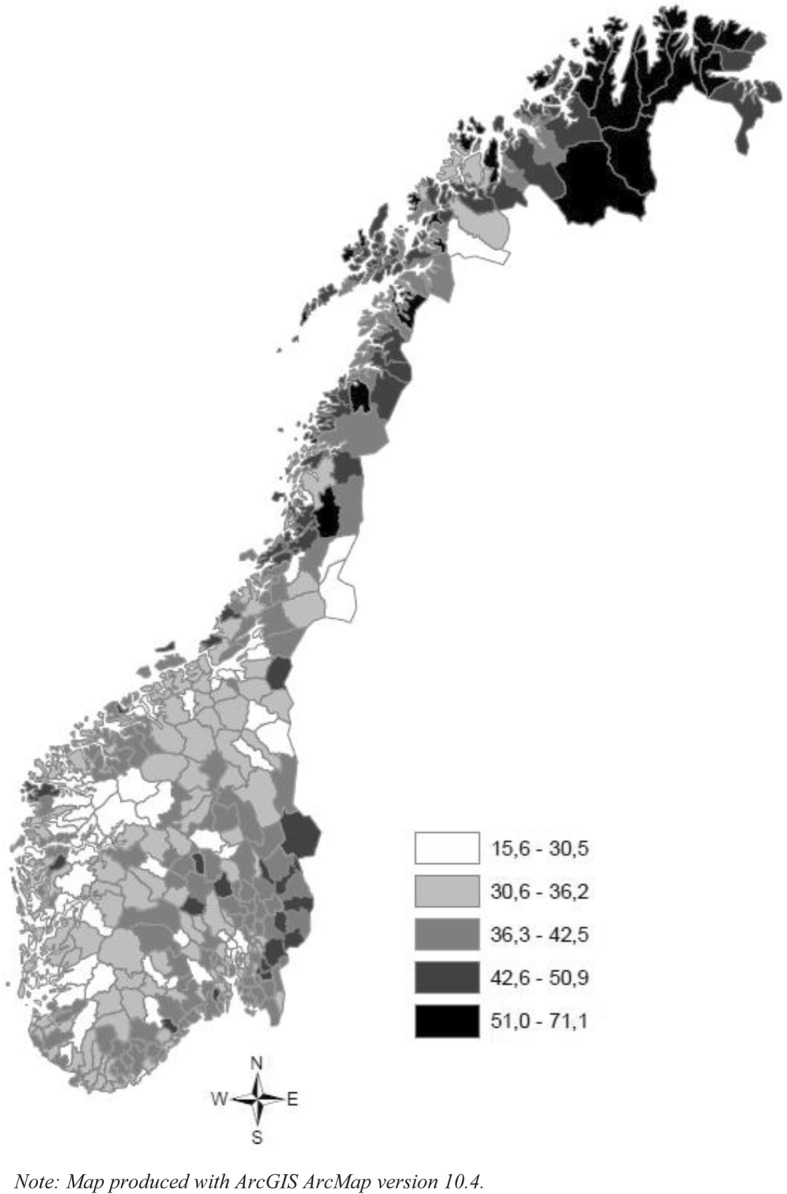
The geographic distribution of non-completers of upper secondary education (percentages) among individuals aged 21–40 years (born between 1970 and 1989 in Norway, 2010

**Fig. 3:**
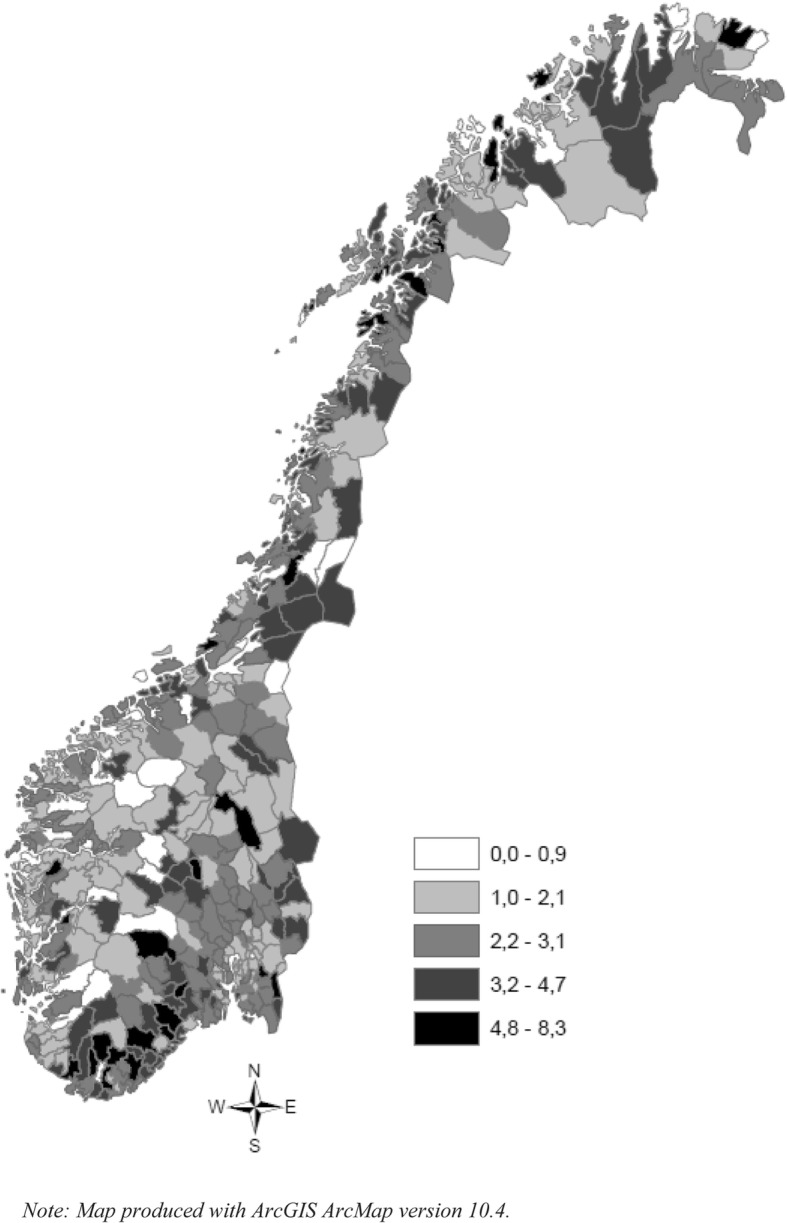
The geographic distributions of disability pensions (percentages) among young adults aged 21–40 years (born between 1970 and 1989 in Norway, 2010

The geographical distribution of secondary education non-completion (Fig. 2) and DP rates (Fig. 3) differs greatly among the Norwegian municipalities. The dropout rates in the 430 municipalities in Norway have a clear geographical pattern (Fig. 2), with high dropout rates in the northern and south-eastern regions, and low dropout rates in western Norway. The prevalence of DP among young adults varies from zero to 8.3%, with an average of 2.0% for the total country.

**Fig. 4:**
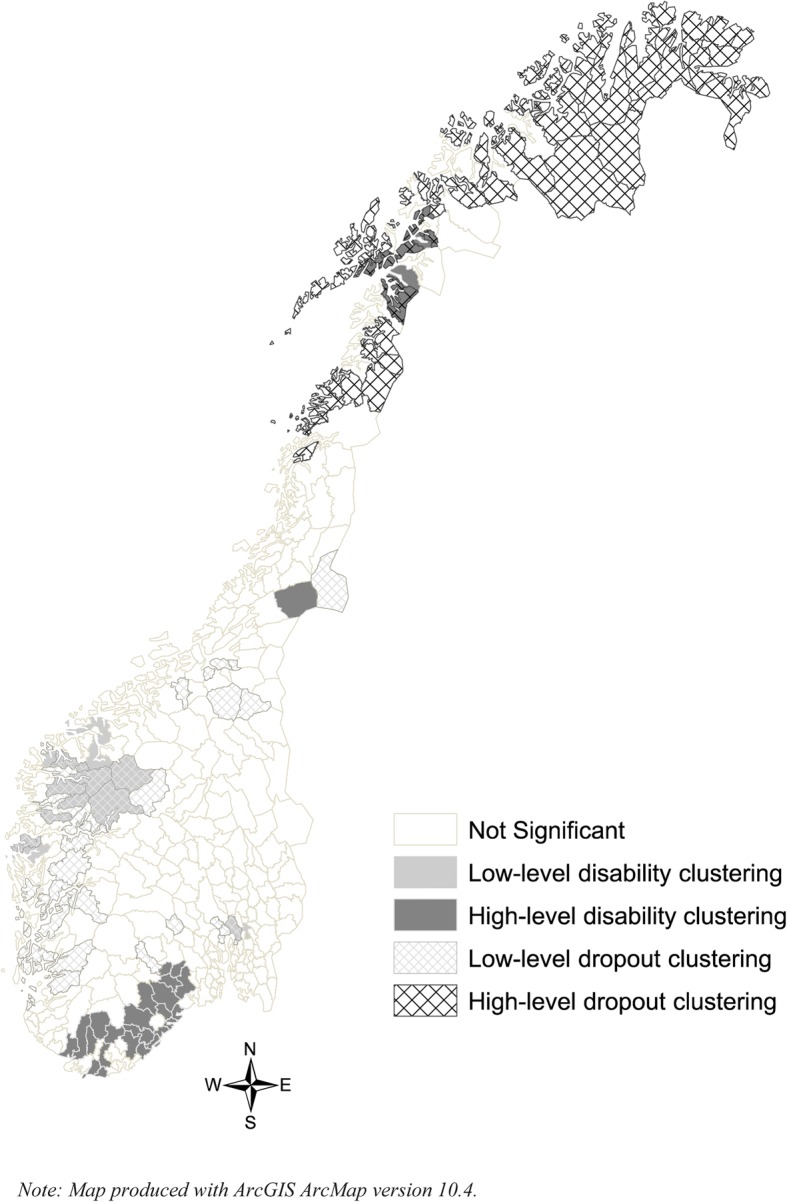
Local indicators of spatial association (LISA) for secondary education non-completion rates and disability pension rates

Measuring the global spatial correlation with the Moran’s I estimator revealed significant clustering in both school non-completion and DP rates, with correlations of .23 and .12 and z-scores of 13.8 and 7.6, respectively. However, comparing Fig. 2 and Fig. 3 revealed distinct geographical distributions of the two groups. The spatial patterns, especially in the northern region, showed a clear clustering of school leavers, but far less clustering of DP rates. The south showed concentrations of municipalities with high DP rates without high dropout rates, while western Norway showed low dropout rates and low DP rates. An analysis of local autocorrelation (LISA) for both non-completion and DP rates confirmed these patterns, as does Fig. 4, which shows the results from both analyses. Specifically, high non-completion rates cluster in much of northern Norway, while clustering of DP rates is very limited here. High DP rates cluster in the southern region, but high non-completion rates do not. Finally, the western region shows substantial overlap in low-rate clustering for both variables.

### Parametric estimation

The prevalence of early DP at the neighbourhood and municipality levels differs. In the first step, we estimated an “empty” model. With only the second and third random intercepts in our model, we found that the ICC are 0.124 and 0.021. In other words, model 1 (in Table 2) suggests that 12.4 and 2.1% of the variation in DP risk can be attributed to differences between neighbourhoods and municipalities, respectively. Table 2 shows the individual and parental covariates of DP receipt in young adulthood. Non-completion of secondary education is positively associated with DP receipt, and this association seems to have strengthened itself over the last two decades. The association between school non-completion and DP receipt is stronger for individuals born in the period 1985–1989 compared to their counterparts born between 1970 and 1974. However, our data do not capture DP receipt after the age of 21–25 for the 1985–89-cohort, which complicates the comparison between the cohorts. Being male, older than average or having parents (mother and/or father) who receive DP are all correlated with higher DP risk before age 40. The interaction term with age and gender are negative and statistical significant, indicating that the positive association between males and DP decreases over time. A complete lack of employment history is associated with the largest DP risk.

**Table 2:** The impact of non-completion of secondary education and its interaction with period of birth on the probability of receiving disability pension (DP)

	Model 1		Model 2		Model 3		Model 4	
	Coef	SE	Coef	SE	Coef	SE	Coef	SE
Fixed effects								
Secondary education non-completion			1.8604***	0.0449	1.8063***	0.0452	1.5831***	0.0464
Cohort								
1970–74			ref		ref		ref	
1975–79			−0.3847***	0.0819	−0.4013***	0.0822	−0.1267	0.0851
1980–84			−0.6735***	0.1243	−0.7075***	0.1246	−0.2871*	0.1322
1985–89			−1.6452***	0.1982	−1.6817***	0.1987	−1.6253***	0.2109
Non-completion*Cohort								
1* 1970–74			ref		ref		ref	
1*1975–79			0.5471***	0.0775	0.5619***	0.0777	0.4528***	0.0799
1* 1980–84			0.7691***	0.0958	0.7825***	0.0960	0.4657***	0.0995
1* 1985–89			1.7037***	0.1528	1.7275***	0.1529	1.2582***	0.1551
Male			0.5092**	01526	0.4742**	0.1532	0.7858***	0.1659
Age			0.0734***	0.0092	0.0737***	0.0092	0.1365***	0.0101
Age*male			−0.0177***	0.0045	−0.0169***	0.0045	−0.0222***	0.0049
Maternal DP								
No					ref		ref	
Yes					0.6020***	0.0318	0.5009***	0.0354
Mother’s identity unknown					0.2322**	0.0808	−0.1565	0.0914
Paternal DP								
No					ref		ref	
Yes					0.4298***	0.0347	0.2996***	0.0388
Father’s identity unknown					−1.6170***	0.1218	−2.5678***	0.1334
No previous employment records							3.7955***	0.0405
Random effects								
Neighbourhood variance (95% CI)	0.4775	0.0289	0.4297	0.0285	0.4319	0.0288	0.3022	0.0283
PCV^a^			−10.0%		−9.6%		−36.7%	
ICC(%)	0.1240	0.0069	0.1133	0.0070	0.1142	0.0070	0.0827	0.0074
MOR	1.93		1.87		1.87		1.69	
Municipality variance (95% CI)	0.0823	0.0129	0.0741	0.0127	0.0619	0.0115	0.0622	0.0119
PCV^a^			−10.0%		−24.8%		−24.4%	
ICC (%)	0.0214	0.0033	0.0195	0.0033	0.0164	0.0030	0.0170	0.0032
MOR	1.32		1.30		1.27		1.27	
-2loglikeliehood	67,825.348		59,195.306		58,211.102		48,854.622	

^a^The proportional change in variance expresses the change in variance at the particular level from the empty model

∗∗∗*p* < 0.001, ∗∗*p* < 0.01, ∗*p* < 0.05

In Table 3, we extended the random intercept logit model for the relationship between the probability of receiving DP and non-completion of secondary education to allow the impact of non-completion to vary across municipalities. The two-level random intercept model, which is nested in the random slope model, is rejected at the 5% significance level (using a likelihood ratio test), suggesting that the impact of school non-completion does vary between municipalities.

**Table 3:** Parameter estimates and log-likelihood values for the random intercept and random slope logistic regression models

	Random intercept		Random slope (coefficient)	
Parameter	Coef	SE	Coef	SE
Individual level				
Intercept	−5.1818***	0.0322	−5.1694***	0.0376
School non-completion	2.3159***	0.0354	2.2977***	0.0364
Municipality level random part				
Residual variance intercept	0.0914	0.0131	0.0941	0.0245
Residual variance slope			0.0337	0.0237
Intercept-slope covariance			−0.0148	0.0208
-2Log likelihood	61,010.002		60,994.002	
BIC	61,048.3		61,045.07	
AIC	61,016		61,002	

***p < 0.001

Likelihood ratio test: LR chi2 = 16.65, *p*-value = 0.0002

Turning to the neighbourhood and municipality variables (Table 4), we found that rural settlement is associated with lower risk of DP in young adulthood. This corresponds well with the patterns observed in Fig. 3, where clusters of some of the country’s highest DP rates are found in the densely populated areas of eastern and southern Norway. The spatial lag variable is positive and significant, indicating that early DP has a regional clustering effect. This confirms the clustering mapped in Fig. 3. Living in municipalities where neighbouring municipalities have high DP rates correlates with higher DP risk, even when adjusting for individual and municipal socioeconomic variables. Models 3–5 (in Table 4) suggest that there is co-variation between the municipal socioeconomic environment and the individual DP risk. However, these associations are conditioned by the completion of secondary education. In other words, the effect of the municipal socioeconomic environment changes dependent on whether or not the individual has completed secondary education. Among non-completers, advantageous municipal socioeconomic conditions, such as high income and education levels and low unemployment and social security payment rates, are all associated with higher DP risk.

**Table 4:** The impact of non-completion of secondary education, municipal socioeconomic factors and their interactions^a^ on the probability of receiving disability pension (DP)

	Model 1		Model 2		Model 3		Model 4		Model 5	
	Coef	SE	Coef	SE	Coef	SE	Coef	SE	Coef	SE
Fixed effects										
Individual level										
Non-completion	2.2416***	0.0329	1.0613*	0.4800	1.3491***	0.2916	2.6458***	0.1449	2.5850***	0.1049
Neighbourhood level										
Rural settlement	−0.3896***	0.0404	−0.3894***	0.0404	−0.3889***	0.0404	−0.3896***	0.0404	−0.3887***	0.0404
Municipality level										
Socioeconomic factors										
Education	0.0226***	0.0052	0.0074	0.0080	0.0221***	0.0052	0.0225***	0.0052	0.0224***	0.0052
Household income	−4.14e-06***	8.95e-07	−4.11e-06***	8.95e-07	−6.20e-06***	1.12e-06	−4.13e-06***	8.96e-07	−4.09e-06***	8.94e-07
Unemployment	−0.0286	0.0309	−0.0285	0.0308	−0.0283	0.0308	0.0760	0.0475	−0.0285	0.0308
Social security benefits	0.0127	0.0107	0.0129	0.0107	0.0130	0.0107	0.0129	0.0108	0.0611***	0.0175
DP spatlag	0.0302**	0.0117	0.0303**	0.0117	0.0306**	0.0117	0.0300*	0.0117	0.0302**	0.0117
Interactions non-completion and municipality factors										
1*Education			0.0177*	0.0072						
1*household income					2.60e-06**	8.47e-07				
1*Unemployment							−0.1248**	0.0433		
1* Social security benefits									−0.0572***	0.0164
General contextual effects										
Neighbourhood variance	0.4214	0.0288	0.4206	0.0288	0.4201	0.0288	0.4213	0.0288	0.4206	0.0288
PCV	−11.8%		−11.9%		−12.0%		−11.8%		−11.9%	
ICC (%)	0.1120	0.0070	0.1118	0.0070	0.1117	0.0070	0.1120	0.0070	0.1118	0.0070
MOR	1.86		1.86		1.86		1.86		1.86	
Municipality variance	0.0503	0.0104	0.0503	0.0104	0.0503	0.0104	0.0503	0.0104	0.0500	0.0103
PCV	−38.9%		−38.9%		−38.9%		−38.9%		−39.2%	
ICC (%)	0.0134	0.0027	0.0134	0.0027	0.0134	0.0027	0.0134	0.0027	0.0133	0.0027
MOR	1.24		1.24		1.24		1.24		1.24	
-2loglikelihood	58,304,708		58,298,74		58,295,14		58,296,46		58,292,728	

^a^adjusted for age, gender and parental DP receipt ^b^The proportional change in variance expresses the change in variance at the particular level from the empty model ∗∗∗*p* < 0.001, ∗∗*p* < 0.01, ∗*p* < 0.05

**Fig. 5:**
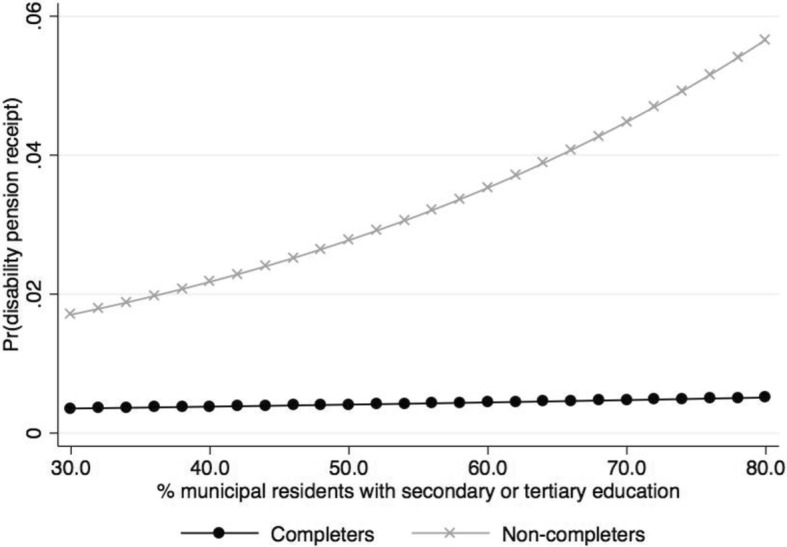
Predictive margins of school completers and non-completers predicting probability (Pr) of receiving DP by percentages of municipal residents with secondary or tertiary education

Keeping other variables constant, the predicted effects of the municipal socioeconomic variables, such as education level, can be evaluated by adding together the municipal education level (percentages of inhabitants with secondary or tertiary education) and school non-completion term and their interactions after filling in for different levels of municipal education (i.e. percentages with secondary or tertiary education). Doing this reveals that the probability of receiving a DP among school non-completers increases with increasing level of municipal education level, whereas among school completers the probability is more or less constant (< 0.5%) regardless of education level (Fig. 5). Among non-completers residing in a municipality with 60% of inhabitants with secondary or tertiary education the risk is 3.5%, and in a municipality with 75% the risk has increased to about 5%.

## Discussion

This study examined how medically based DP among young adults varies geographically, and how municipal socioeconomic conditions interact with non-completion of secondary education in determining DP risk. Findings from the current study reinforce the relevance of the residential context in DP risk among young adults. In support of previous studies, we found that non-completers of secondary education are more likely to receive early DP than completers. Our parametric estimation, however, suggest that the association between school non-completion and DP receipt varies across municipalities. The key contributions of this study are related to the exploration of how different municipal socioeconomic conditions interact with non-completion of secondary education, to alter DP risk in young adulthood. Municipalities with high socioeconomic profiles are, in general, associated with both a lower risk of non-completion and DP receipt [36–38, 60]. But this association does not hold for all groups. We found that non-completion has a stronger association with DP in socioeconomically advantaged municipalities. In other words, living in a high-status municipality (i.e. high income, high education levels and low unemployment and social security payment rates) is associated with higher risk of DP among those who do not complete their secondary education.

Spatial clustering of DPs, which is evident at the municipality level, can be interpreted in light of Wilson’s [61] assumption that neighbourhood characteristics influence collective socialisation processes by shaping the type of role models youth are exposed to outside their homes. Interactions with community peers and adults shape the norms, values, aspirations and, ultimately, the behaviours of the residents. Hence, advantaged neighbourhoods, where most adults have attained advanced formal education and steady jobs, will foster behaviours and attitudes within the next generations that are conducive to success in both education and work. In less advantaged communities, where the share of the population participating in the labour force is low and the dependency on welfare benefits is high, the positive attitudes toward education and work career may be less common. Rege, et al. [62] suggest that being disability may have “contagion” effect in the community, meaning that the propensity to receive DP increases when many people around you also depend on DP. Community characteristics represent more than the sum of their parts. Socioeconomic factors at the level of individuals may fail to protect even the health of well-off people if they live in socioeconomically disadvantaged neighbourhoods [35], and socioeconomically privileged neighbourhoods may impose an added health risk to the marginalised.

Not only did we find that municipal factors were correlated with young inhabitants’ DP risk, but we also uncovered regional effects. The DP rates in neighbouring municipalities are significantly associated with DP risk, suggesting inter-municipal processes. Most municipalities are embedded within larger regional contexts, and common historical, political, economic, and cultural factors shape them. Todd [63] suggests that the inherited regional differences in social structure affect our practices and values, which, in turn, will dispose us to think and act institutionally. In western Norway, a region with traditional Christian orthodoxy [64], people strongly value education and express this value by attaining higher formal education. Official statistics show that the population of western Norway, in general, has better health and has the highest life expectancy in the country [65]. Thus, our finding of high secondary education completion rates and low DP prevalence in western Norway is not surprising. Like Markussen, et al. [66], we found that northern Norway has a higher non-completion rate than any other region. Yet, this region has no systematic clustering of high DP prevalence. Even outside of our focal age group, the education level here is relatively low and many employers have traditionally not required a secondary education.

In line with previous Norwegian population studies [10, 11], we found a strong association between school non-completion and DP risk in young adulthood. This association may emerge from risk factors not directly modelled. Poor health in adolescence is, for example, strongly associated with school non-completion [67]. Such health problems may, indeed, lower the chances of finding a job and increase the probability of receiving DP in young adulthood. Our analysis, however, show that the relationship between school non-completion and DP risk varies between municipalities and that the municipal socioeconomic environment has a substantial impact on this relationship. Previous Nordic population studies have demonstrated a relationship between municipal socioeconomic conditions and DP prevalence [36–38]. What stands out in our study is our finding that living in municipalities with high education and income levels and low unemployment and social security benefit rates was associated with higher DP risk among non-completers. Advantageous socioeconomic conditions are generally associated with increased individual probability of both completing secondary education and successfully entering the labour market [32, 38]. Nevertheless, population and community characteristics can, indeed, interact with individual characteristics [68] and, have differing impacts across population groups. Non-completers in areas with a lower education level may, according to the relative deprivation hypothesis [34, 44], be less at risk than non-completers in more socioeconomically advanced areas. Mishra and Carleton [69] demonstrated that subjective feelings of relative deprivation are linked with poorer physical and mental health. Moreover, social distance, distrust and lack of cohesion between population groups often characterise communities with high material and social inequalities [70]. This may lead to higher stress levels, especially for those at the bottom of the social ladder, resulting in higher prevalence of stress-related morbidity and health risk behaviours [71, 72]. Moreover, young adults without a secondary school degree may face greater difficulties in the labour market in societies with ample access to highly qualified applicants. Disparities between workers’ resources and structural features of the job market may negatively impact their health [73]. School non-completion may, in other words, be more detrimental and contribute to stronger health selection in socioeconomically advanced areas. Hence, municipalities with seemingly strong socioeconomic profiles pose added risks for disadvantaged young adults without a secondary school degree. Similar to Reime and Claussen [37], we found that rural settlement was associated with a lower risk of DP among young school leavers. The education level in rural parts of Norway is generally lower compared to more urban areas, with easier access to jobs not requiring a formal education.

Based on the relatively high portion of young people receiving DP in Norway, one might question how DPs are granted. Disability benefits granted to young people are a substitute for lost income due to disability. In order to be entitled as a young disabled person, one must be under 26 years old upon becoming seriously and permanently ill, and said illness must be clearly documented by a medical doctor/specialist. The causes of the increasing proportion of disabled young people in Norway are, however, highly complex and unclear [3]. It is primarily mental illness that causes young people to become disabled. One important explanation is likely that it has become more difficult for young people with mental illnesses to obtain and retain employment [3]. Another explanation of this growth is tied to changes in social security schemes and expectations related to receiving valuable welfare schemes. As time-limited disability benefits were replaced with work-disbursement benefits in 2010, many were transferred to this new benefit. Today, about 70% of those who received temporary disability benefits have been granted DP [74].

A major strength of our study is the use of large, nationally representative registry data with multiple explanatory factors at the individual, family and neighbourhood levels linked to population-based municipal socioeconomic factors. The use of high quality, official longitudinal registry data covering almost the entire Norwegian population greatly minimises the risk of selection bias and random errors in our analyses. However, using such a large dataset introduces the risk of identifying significant, but inconsequential effects [75]. Although studies based on large sample size have many advantages, marginally significant effects observed in such studies typically mean that the predictive effect of the exposure is quite modest [75]. Moreover, a large dataset with multiple explanatory variables introduces the risk of over-adjusting for inter-level confounding, in that contextual factors might determine some of the individual level variables [76]. We limited the risk of over-adjustment by including only a small number of individual and family level predictors that previous research has shown to affect DP risk. Our study has several limitations. First, there are many methodological challenges in the analysis of neighbourhood contextual effects, such as identification of the appropriate boundaries [77], endogeneity [78], structural confounding [79] and excessive extrapolation in multilevel modelling [78]. An obvious challenge related to the estimation of neighbourhood effects in Norway is the major differences in population density between the different regions of the country. A 30% random and stratified sample of the population leads to a small number of study participants in a significant proportion of neighbourhoods. Moreover, the issue with selective residential mobility poses interpretational challenges. For instance, advantageous socioeconomic circumstances and healthier individuals tend to move to or remain in less deprived neighbourhoods [80]. Finally, an ideal study should include longitudinal explanatory data at multiple appropriate levels and allow the levels (i.e. the context) to change over time. However, our data do not allow us to control for this and, thus, prevent the adoption of this analytic framework.

## Conclusions

This study underlines the importance of completing secondary education in the prevention of medically based DP among young adults in modern society. However, the study also demonstrates the significance of the residential context and local socioeconomic environment in individual variation in DP receipt. Low educational achievement and DP receipt have several central determinants in common, but comparing the geographical distributions of non-completion and DP rates reveal regional divergence. The risk factors manifest themselves at different structural levels, and a risk measured at the individual level may have a different effect when evaluated at the municipal level. This creates divergence in the geographical distribution of non-completion and DP rates, and anything but a multilevel analysis would likely conflate these results. Moreover, this study suggests that the population under study largely defines the relationship between risk factors and early DP. Advantageous municipal socioeconomic conditions will, in general, increase both the individual probability of completing secondary education and successfully entering the labour market. However, among non-completers, these municipal conditions are associated with a higher risk of receiving DP in young adulthood. Furthermore, living in rural communities lowers the risk of early DP. The mostly rural northern Norway has the highest non-completion rates in the country without particularly high levels of DP. These communities offer relatively well-paid jobs in the maricultural and fishing industry that do not require high formal education. Young adults with no previous employment records have the highest risk of receiving DP. Young people who do not finish secondary education are more marginalised in societies that place a higher weight on formal education. As more students complete their education, the potential marginalisation and barriers into the job market for those who drop out increases. As our results suggest, environmental factors are important determinants of risk, and measures aimed at lowering DP rates will probably fail to reach their potential without an understanding of the risks posed by the local environment. Future efforts to promote social equality and successful transitions to adulthood with regard to work and health should focus on the interplay between the local community and individual factors.

## References

[1] OECD. Sickness, disability and work: breaking the barriers: a synthesis of findings across OECD countries. Paris: OECD Publishing; 2010. 10.1787/9789264088856-en.

[2] Ellingsen J. Utviklingen i uføretrygd1 per 31. Mars 2017 [in Norwegian]. Oslo: Norwegian Labour and Welfare Administration (NAV); 2017. https://www.nav.no/no/NAV+og+samfunn/Statistikk/AAP+nedsatt+arbeidsevne+og+uforetrygd+-+statistikk/Uforetrygd/Uforetrygd+-+Statistikknotater

[3] Brage S, Thune O. Ung uførhet og psykisk sykdom [In Norwegian]. Oslo: Arbeid og Velferd: Norwegian Labour and Welfare Administration (NAV), 2015:37–49.

[4] Dølvik J, Fløtten T, Hippe J, Jordfald B (2015). The Nordic model towards 2030. A new chapter?.

[5] OECD. Mental health and work. Paeris: OECD Publishing; 2013. 10.1787/9789264178984-en

[6] Avendano M, Berkman LF. Labor markets, employment policies, and health. In: Berkman L, Kawachi I,Glymour M, eds. Social epidemiology Second ed. New York: Oxford University Press. 2014:182–233.

[7] Bartley M, Ferrie J, Montgomery SM (2009). Health and labour market disadvantage: unemployment, non-employment, and job insecurity. Social determinants of health.

[8] Claussen B (1998). Restricting the influx of disability beneficiaries by means of law: experiences in Norway. Scand J Public Health.

[9] Viner RM, Ozer EM, Denny S, et al. Adolescence and the social determinants of health. Lancet. 2012;379(9826):1641–1652. 10.1016/S0140-6736(12)60149-4. 10.1016/S0140-6736(12)60149-4 22538179

[10] Gravseth HM, Bjerkedal T, Irgens LM, Aalen OO, Selmer R, Kristensen P (2007). Life course determinants for early disability pension: a follow-up of Norwegian men and women born 1967-1976. Eur J Epidemiol.

[11] De Ridder KA, Pape K, Johnsen R, Westin S, Holmen TL, Bjorngaard JH (2012). School dropout: a major public health challenge: a 10-year prospective study on medical and non-medical social insurance benefits in young adulthood, the young-HUNT 1 study (Norway). J Epidemiol Community Health.

[12] Bäckman O, Jakobsen V, Lorentzen T, Österbacka E, Dahl E (2015). Early school leaving in Scandinavia: extent and labour market effects. J Eur Soc Policy.

[13] Bäckman O, Jakobsen V, Lorentzen T, Österbacka E, Dahl E. Dropping out in Scandinavia social exclusion and labour market attachment among upper secondary school dropouts in Denmark, Finland, Norway and Sweden: Institute for Futures Studies; 2011.

[14] OECD. Education at a glance 2014: OECD Indicators: OECD Publishing. p. 2014. 10.1787/eag-2017-en

[15] Bäckman O, Nilsson A. Pathways to social exclusion—a life-course study. Eur Sociol Rev. 2010;27(1):107–23. 10.1093/esr/jcp064.

[16] Caspi A, Wright BRE, Moffitt TE, Silva PA (1998). Early failure in the labor market: childhood and adolescent predictors of unemployment in the transition to adulthood. Am Sociol Rev.

[17] Marmot MG, Bell R (2012). Fair society, healthy lives. Public Health.

[18] Marmot MG, Wilkinson RG (2006). Social determinants of health.

[19] Myhr A, Haugan T, Espnes GA, Lillefjell M. Disability pensions among young adults in vocational rehabilitation. J Occup Rehabil. 2015;26(1): 95–102. 10.1007/s10926-015-9590-5. 10.1007/s10926-015-9590-5 26141951

[20] OECD. Society at a Glance 2016: OECD Social Indicators. Paris: OECD Publishing; 2016. 10.1787/9789264261488-en

[21] Amone-P’olak K, Burger H, Huisman M, Oldehinkel AJ, Ormel J (2011). Parental psychopathology and socioeconomic position predict adolescent offspring's mental health independently and do not interact: the TRAILS study. J Epidemiol Community Health.

[22] Boe T, Sivertsen B, Heiervang E, Goodman R, Lundervold AJ, Hysing M. Socioeconomic status and child mental health: the role of parental emotional well-being and parenting practices. J Abnorm Child Psychol. 2013;42(5): 705–15. 10.1007/s10802-013-9818-9. 10.1007/s10802-013-9818-9 24150864

[23] Boe T, Overland S, Lundervold AJ, Hysing M (2012). Socioeconomic status and children's mental health: results from the Bergen child study. Soc Psychiatry Psychiatr Epidemiol.

[24] Galobardes B, Lynch JW, Smith GD (2008). Is the association between childhood socioeconomic circumstances and cause-specific mortality established? Update of a systematic review. J Epidemiol Community Health.

[25] Chen E, Martin AD, Matthews KA (2006). Socioeconomic status and health: do gradients differ within childhood and adolescence?. Soc Sci Med.

[26] Repetti RL, Taylor SE, Seeman TE (2002). Risky families: family social environments and the mental and physical health of offspring. Psychol Bull.

[27] Poulton R, Caspi A, Milne BJ (2002). Association between children's experience of socioeconomic disadvantage and adult health: a life-course study. Lancet.

[28] Davis-Kean PE (2005). The influence of parent education and family income on child achievement: the indirect role of parental expectations and the home environment. J Fam Psychol.

[29] Myhr A, Lillefjell M, Espnes GA, Halvorsen T (2017). Do family and neighbourhood matter in secondary school completion? A multilevel study of determinants and their interactions in a life-course perspective. PLoS One.

[30] Pape K, Bjorngaard JH, De Ridder KA, Westin S, Holmen TL, Krokstad S (2013). Medical benefits in young Norwegians and their parents, and the contribution of family health and socioeconomic status. The HUNT study, Norway. Scand J Public Health.

[31] Harkonmaki K, Korkeila K, Vahtera J (2007). Childhood adversities as a predictor of disability retirement. J Epidemiol Community Health.

[32] Nieuwenhuis J, Hooimeijer P, Meeus W (2015). Neighbourhood effects on educational attainment of adolescents, buffered by personality and educational commitment. Soc Sci Res.

[33] Reijneveld SA (2002). Neighbourhood socioeconomic context and self reported health and smoking: a secondary analysis of data on seven cities. J Epidemiol Community Health.

[34] Stafford M, Marmot M (2003). Neighbourhood deprivation and health: does it affect us all equally?. Int J Epidemiol.

[35] Stafford M, McCarthy M, Marmot MG, Wilkinson RG (2006). Neighbourhoods, housing, and health. Social determinants of health.

[36] Laaksonen M, Gould R. The effect of municipality characteristics on disability retirement. Eur J Public Health. 2013;24(1):116–21. 10.1093/eurpub/ckt129 24025665

[37] Reime LJ, Claussen B (2013). Municipal unemployment and municipal typologies as predictors of disability pensioning in Norway: a multilevel analysis. Scand J Public health.

[38] Krokstad S, Magnus P, Skrondal A, Westin S (2004). The importance of social characteristics of communities for the medically based disability pension. Eur J Pub Health.

[39] Kavanagh AM, Goller JL, King T, Jolley D, Crawford D, Turrell G (2005). Urban area disadvantage and physical activity: a multilevel study in Melbourne, Australia. J Epidemiol Community Health.

[40] Subramanian SV, Kawachi I, Kennedy BP. Does the state you live in make a difference? Multilevel analysis of self-rated health in the US. Soc Sci Med. 2001;53(1):9–19. 10.1016/S0277-9536(00)00309-9. 10.1016/s0277-9536(00)00309-9 11380164

[41] Browning CR, Cagney KA (2002). Neighborhood structural disadvantage, collective efficacy, and self-rated physical health in an urban setting. J Health Soc Behav.

[42] Marinacci C, Spadea T, Biggeri A, Demaria M, Caiazzo A, Costa G (2004). The role of individual and contextual socioeconomic circumstances on mortality: analysis of time variations in a city of north West Italy. J Epidemiol Community Health.

[43] Wilkinson RG, Marmot MG, Wilkinson RG (2006). Ourselves and others - for better or worse: social vulnerability and inequality. Social determinants of health.

[44] Wilkinson RG. Ourselves and others–for better or worse: social vulnerability and inequality. In: Marmot MG, Wilkinson RG, eds. Social Determinants of Health. Oxford Oxford University Press. 2006:341–57.

[45] Akselsen A, Lien S, Siverstøl Ø (2007). FD-Trygd, list of variables.

[46] Ellingsen J. Utviklingen i uføretrygd per 31. mars 2018 [in Norwegian]: The Norwegian Labour and Welfare Administration; 2018. https://www.nav.no/no/NAV+og+samfunn/Statistikk/AAP+nedsatt+arbeidsevne+og+uforetrygd+-+statistikk/Uforetrygd/

[47] Statistics Norway. Population and area in urban settlements 2016 [In Norwegian]. [Official statistics ]. https://www.ssb.no/befolkning/statistikker/beftett/aar.

[48] Goldstein H (1995). Multilevel statistical models.

[49] Rabe-Hesketh S, Skrondal A., ed. Multilevel and longitudinal modeling using Stata, Volume II: Categorical responses, counts, and survival. Third ed. Texas: STATA press; 2012.

[50] Snijders TAB, Bosker RJ. Multilevel analysis: an introduction to basic and advanced multilevel modeling, second ed. London: Sage Publishers; 2012.

[51] Merlo J, Chaix B, Yang M, Lynch J, Råstam L (2005). A brief conceptual tutorial of multilevel analysis in social epidemiology: linking the statistical concept of clustering to the idea of contextual phenomenon. J Epidemiol Community Health.

[52] Larsen K, Merlo J (2005). Appropriate assessment of neighborhood effects on individual health: integrating random and fixed effects in multilevel logistic regression. Am J Epidemiol.

[53] Merlo J, Viciana-Fernández FJ, Ramiro-Fariñas D (2012). Population RGotLDotA. Bringing the individual back to small-area variation studies: a multilevel analysis of all-cause mortality in Andalusia, Spain. Soc Sci Med.

[54] Browne WJ, Subramanian SV, Jones K, Goldstein H (2005). Variance partitioning in multilevel logistic models that exhibit overdispersion. J R Stati Soc: A (Stat Soc).

[55] Merlo J, Chaix B, Ohlsson H (2006). A brief conceptual tutorial of multilevel analysis in social epidemiology: using measures of clustering in multilevel logistic regression to investigate contextual phenomena. J Epidemiol Community Health.

[56] Chaix B, Leyland AH, Sabel CE (2006). Spatial clustering of mental disorders and associated characteristics of the neighbourhood context in Malmö, Sweden, in 2001. J Epidemiol Community Health.

[57] Chaix B, Merlo J, Subramanian S, Lynch J, Chauvin P (2005). Comparison of a spatial perspective with the multilevel analytical approach in neighborhood studies: the case of mental and behavioral disorders due to psychoactive substance use in Malmö, Sweden, 2001. Am J Epidemiol.

[58] Anselin L (1995). Local indicators of spatial association—LISA. Geogr Anal.

[59] Anselin L, Getis A (1992). Spatial statistical analysis and geographic information systems. Ann Reg Sci.

[60] Ainsworth JW (2002). Why does it take a village? The mediation of neighborhood effects on educational achievement. Soc Forces.

[61] Wilson WJ (2011). When work disappears: The world of the new urban poor: vintage.

[62] Rege M, Telle K, Votruba M (2012). Social interaction effects in disability pension participation: evidence from plant downsizing*. Scand J Econ.

[63] Todd E (1987). The causes of progress. Culture, authority and change.

[64] Rokkan S, Lipset SM, Rokkan S (1967). Geography, religion and social class: crosscutting cleavages in Norwegian politics. Party systems and voter alignments: cross-national perspectives.

[65] Norwegian Institute of Public Health. Oslo: Public health report: Life expectancy in Norway; 2016. https://www.fhi.no/en/op/hin/befolkning-og-levealder/levealderen-i-norge/

[66] Markussen E, Lødding B, Holen S (2012). De’ hær e’kke nokka for mæ [in Norwegian].

[67] De Ridder KA, Pape K, Johnsen R, Holmen TL, Westin S, Bjorngaard JH (2013). Adolescent health and high school dropout: a prospective cohort study of 9000 Norwegian adolescents (the young-HUNT). PLoS One.

[68] Shouls S, Congdon P, Curtis S (1996). Modelling inequality in reported long term illness in the UK: combining individual and area characteristics. J Epidemiol Community Health.

[69] Mishra S, Carleton RN (2015). Subjective relative deprivation is associated with poorer physical and mental health. Soc Sci Med.

[70] Kawachi I, Kennedy BP, Lochner K, Prothrow-Stith D (1997). Social capital, income inequality, and mortality. Am J Public Health.

[71] Wilkinson RG (1999). Health, hierarchy, and social anxiety. Ann N Y Acad Sci.

[72] Wilkinson RG (1999). Income inequality, social cohesion, and health: clarifying the theory—a reply to Muntaner and lynch. Int J Health Serv.

[73] Dahl E, Elstad JI (2001). Recent changes in social structure and health inequalities in Norway. Scand J Public Health Suppl.

[74] Kann IC, Kristoffersen P. Arbeidsavklaringspenger – et venterom for uførepensjon? [In Norwegian]. Arbeid og velferd: Norwegian Labour Welfare Administration (NAV). 2014;2:101–15.

[75] Kaplan RM, Chambers DA, Glasgow RE (2014). Big data and large sample size: a cautionary note on the potential for Bias. Clin Transl Sci.

[76] Lindström M, Merlo J, Östergren P-O (2003). Social capital and sense of insecurity in the neighbourhood: a population-based multilevel analysis in Malmö, Sweden. Soc Sci Med.

[77] Merlo J, Ohlsson H, Lynch KF, Chaix B, Subramanian S (2009). Individual and collective bodies: using measures of variance and association in contextual epidemiology. J Epidemiol Community Health.

[78] Oakes JM (2004). The (mis) estimation of neighborhood effects: causal inference for a practicable social epidemiology. Soc Sci Med.

[79] Messer LC, Oakes JM, Mason S (2010). Effects of socioeconomic and racial residential segregation on preterm birth: a cautionary tale of structural confounding. Am J Epidemiol.

[80] Norman P, Boyle P, Rees P (2005). Selective migration, health and deprivation: a longitudinal analysis. Soc Sci Med.

